# Oregano Essential Oil in Livestock and Veterinary Medicine

**DOI:** 10.3390/ani14111532

**Published:** 2024-05-22

**Authors:** Huan Cui, Cheng Zhang, Kai Su, Tingli Fan, Ligong Chen, Zitong Yang, Mingda Zhang, Jiaqi Li, Yuxin Zhang, Juxiang Liu

**Affiliations:** 1 College of Veterinary Medicine, Hebei Agricultural University, Baoding 071000, China; huancui1349@hebau.edu.cn (H.C.); chengzhang1349@hebau.edu.cn (C.Z.); clg01@163.com (L.C.); yang5241225@163.com (Z.Y.); 19832229138@163.com (M.Z.); ljq15027874688@163.com (J.L.); 15081207171@163.com (Y.Z.); 2 Department of Agricultural and Animal Husbandry Engineering, Cangzhou Technical College, Cangzhou 061000, China; sukai0324@126.com (K.S.); ftl2015@126.com (T.F.)

**Keywords:** oregano essential oil, antibacterial, antiviral, antioxidant, anti-inflammatory

## Abstract

**Simple Summary:**

Simple Summary: Carvacrol and thymol are the primary constituents of oregano essential oil (OEO) and possess significant antibacterial, antiviral, and antifungal properties. Recent research has showcased the potent antioxidant, anti-inflammatory, antidiabetic, and cancer-inhibiting effects of OEO. The properties of OEO hold potential implications for the livestock and veterinary industries. This manuscript aims to evaluate the utility of OEO in the domains of animal husbandry and veterinary medicine.

**Abstract:**

With a growing global concern over food safety and animal welfare issues, the livestock and veterinary industries are undergoing unprecedented changes. These changes have not only brought challenges within each industry, but also brought unprecedented opportunities for development. In this context, the search for natural and safe products that can effectively replace traditional veterinary drugs has become an important research direction in the fields of animal husbandry and veterinary medicine. Oregano essential oil (OEO), as a natural extract, is gradually emerging in the fields of animal husbandry and veterinary medicine with its unique antibacterial, antioxidant, and multiple other biological activities. OEO not only has a wide antibacterial spectrum, effectively fighting against a variety of pathogenic microorganisms, but also, because of its natural properties, helps us to avoid traditional veterinary drugs that may bring drug residues or cause drug resistance problems. This indicates OEO has great application potential in animal disease treatment, animal growth promotion, and animal welfare improvement. At present, the application of OEO in the fields of animal husbandry and veterinary medicine has achieved preliminary results. Studies have shown that adding OEO to animal feed can significantly improve the growth performance and health status of animals and reduce the occurrence of disease. At the same time, pharmacokinetic studies in animals show that the absorption, distribution, metabolism, and excretion processes of OEO in animals shows good bioavailability. In summary, oregano essential oil (OEO), as a substitute for natural veterinary drugs with broad application prospects, is gradually becoming a research hotspot in the field of animal husbandry and veterinary medicine. In the future, we look forward to further tapping the potential of OEO through more research and practice and making greater contributions to the sustainable development of the livestock and veterinary industries.

## 1. Introduction

In recent years, there has been a notable increase in population, prompting a heightened focus on augmenting food production. Antimicrobial growth promoters are extensively employed in veterinary medicine to treat and prevent animal diseases and are incorporated into animal feed to enhance productivity [1]. Although the precise mechanism through which antimicrobial growth promoters stimulate animal growth remains inadequately understood, investigations conducted on germ-free chickens have demonstrated that the growth-promoting effects of antimicrobial agents are mediated by their antimicrobial properties. The following hypotheses have been previously proposed: (1) antimicrobial growth promoters can safeguard nutrients against bacterial deterioration; (2) antimicrobial growth promoters can facilitate the absorption of nutrients within the intestinal tract; (3) antimicrobial growth promoters can enhance the composition of intestinal microorganisms and mitigate the production of toxins by intestinal bacteria; and (4) antimicrobial growth promoters can diminish the occurrence of intestinal diseases [2,3,4].

In animal husbandry, antibiotic growth promoters are widely added to animal feed to accelerate the growth rate of livestock. However, this over-reliance has caused serious problems. The abuse of antibiotics causes the microbial population in animals to develop strong antibiotic resistance, which not only threatens the health of animals, but also increases the risk of transferring antibiotic resistance genes to human microbiota [5]. This cross-species spread of drug resistance poses a huge threat to global public health. In light of this, many countries have moved to completely ban the use of antibiotics in animal feed [6]. However, the ban has also had a knock-on effect, with the growth rate of animals slowing down and consumers at increased risk of foodborne infections due to an increase in livestock infection rates [7]. This situation urgently requires us to find alternative animal farming solutions to ensure the sustainable development of animal husbandry and public health safety. Antimicrobial resistance has been a growing problem in human medicine over the past 80 years. This can be attributed to the excessive and inappropriate use of antibiotics, as well as the wide range of antimicrobial agents utilized in different countries [8,9,10]. Since the legal ban on the use of antimicrobial growth promoters in animal feed, the livestock industry has faced enormous challenges, such as slowed animal growth, increased disease rates, and declining farming profits. Consequently, there is an immediate necessity to explore novel and more efficient alternatives [11]. Plant-derived oregano essential oil (OEO) has attracted worldwide attention due to its antibacterial, anti-inflammatory, and intestinal microbiota homeostasis effects, and this essential oil may replace antimicrobial growth promoters with a large and profound beneficial impact on the livestock industry [12]. The application of OEO in animal husbandry and production is very extensive, and the addition of oregano in piglets, broilers, cows, rabbits, and aquatic animal feed has been reported. Oregano oil compound nano emulsion has obvious therapeutic effect on acute endometritis of clinical sows [13]. Oregano essential oil can inhibit methane production in rumen of beef cattle [14]. Adding oregano oil to feed can improve the performance of rabbits, chickens and dairy cows [15,16,17,18].

## 2. Essential Oil Composition of Oregano Species

The botanical name of oregano is *Origanum majorana* L. Oregano is a perennial flowering plant of the Lamiaceae family that is widely used in traditional medicine for the treatment of a variety of diseases (parasites, hypertension, respiratory infections, diabetes, and many others) in addition to its flavoring agent [19,20,21]. Ramadan et al. divided oregano into four distinct groups: Greek oregano (*Origanum vulgare*), Turkish oregano (*Origanum onites*), Spanish oregano (*Coridohymus capitatu*), and Mexican oregano (*Lippia graveolens*) [22]. Oregano has been studied as a source of bioactive compounds that are rich in polyphenolic compounds and secondary metabolites produced by plants and that have antioxidant properties. Therefore, it has been extensively studied for its potential as an antioxidant, antibacterial, antifungal, anti-inflammatory, and skin defense aid [23,24,25,26].

The composition of OEO is highly intricate, consisting primarily of a diverse array of terpenes, phenols, phenolic acids, and other compounds, with carvacrol and thymol being the predominant constituents. Additionally, p-cymene, caryophyllene oxide, β-caryophyllene, γ-terpinene, α-humelene, terpinene-4-ol, linalool, and various other compounds are present [27,28,29,30] (Table 1). The composition of OEO varies greatly from place to place, from time to time, and in its different parts (Table S1). The biological efficacy of OEO is intimately linked to its phytochemical characteristics [31,32,33,34,35,36].

## 3. Bioactivity of Essential Oils in Oregano Species

### 3.1. Anti-Inflammatory Activity of Essential Oils in Oregano Species

Inflammation is a prevalent occurrence in both acute and chronic debilitating diseases and is a fundamental component of numerous physiological and pathological processes [37,38]; it is considered a natural response of the body’s self-protective mechanisms. It is widely acknowledged that upon production and release from diverse cell types, cytokines react to inflammatory stimulation, connecting cellular damage to inflammatory responses and facilitating the progression of inflammation [39,40]. Inflammation is a dynamic phenomenon characterized by the involvement of proinflammatory cytokines, including tumor necrosis factor (TNF)-α, interleukin (IL)-1β, and vascular endothelial growth factor (VEGF), which play pivotal roles in the event of tissue death or external stimulation. This process encompasses a multifaceted network of numerous mediators, diverse cellular components, and multiple pathways [41,42].

OEO derived from oregano exhibits notable inhibitory effects on cell proliferation and inflammation by reducing the expression levels of inflammatory biomarkers, including monocyte chemokine (MCP-1), intracellular cell adhesion molecule 1 (ICAM-1), and vascular cell adhesion molecule 1 (VCAM-1) [43]. Investigations into the phenolic compounds, terpenes, and substance composition and biological activity of oregano mexicana revealed that the phenolic and terpene extracts exhibited notable inhibitory effects on the production of reactive oxygen species (ROS) and nitric oxide (NO), as well as on the mitochondrial activity of LPS-induced RAW 264.7 macrophage inflammation. These findings suggest that OEO possesses anti-inflammatory properties [44].

To further explore the mechanism by which OEO inhibits inflammation, Villarreal et al. evaluated the effect of carvacrol on a mouse model of paw inflammation induced by complete Freund’s adjuvant [45]. They found that carvacrol possesses anti-inflammatory properties in a mouse model of inflammation. This is achieved through the downregulation of proinflammatory mediators, the promotion of anti-inflammatory cytokines (IL-10), and subsequent inhibition of IL-1β and PGE2 expression. In a study evaluating the effects of carvacrol intervention on liver injury in type 2 diabetic mice, Li et al. reported significant reductions in the serum levels of TC, TG, LDL-C, ALT, and AST after a 2-week carvacrol intervention, while HDL-C levels significantly increased [46]. Chen et al. evaluated the effect of carvacrol on vascular inflammation in diabetic mice and reported that carvacrol does more than just alleviate vascular endothelial cell damage [47]. In in vitro experiments, carvacrol inhibited hyperglucose-induced endothelial cell dysfunction by promoting vascular endothelial cell apoptosis and inhibiting cell viability. These findings suggest that carvacrol can alleviate endothelial dysfunction and vascular inflammation in T2DM patients. Rupasinghe et al. evaluated the anti-inflammatory efficacy of carvacrol in vitro using an in vitro model of *Streptococcal pharyngitis* induced by human tonsillar epithelial cells (HTonEpiCs) of *Streptococcus pyogenes* cell wall antigens [48]. It was found that carvacrol inhibits the production of proinflammatory mediators such as IL-6, IL-8, HBD-2, GCP-2, ENA-78, PGE2, and COX-2 to improve pain associated with *Streptococcal pharyngitis*, and further research into the use of carvacrol as a natural health additive can continue. Ma et al. explored the mechanism by which carvacrol protects mice from LPS-induced sepsis [49]. The results showed that carvacrol significantly improved weight loss in mice with LPS-induced sepsis; ameliorated pathological damage to the liver, lungs, and heart; and attenuated the inflammatory response by inhibiting LPS-induced production of the inflammatory cytokine interleukin-6 (IL-6) in vivo and in vitro. Carvacrol exerts its inhibitory effect on IL-2 production in macrophages primarily via the ERK6/1 signaling pathway. Furthermore, carvacrol has a positive impact on the survival rate of septic mice, revealing its involvement in the pathogenesis of LPS-induced sepsis and its potential as a therapeutic agent for sepsis treatment.

As another major component of OEO, thymol exerts anti-inflammatory effects by inhibiting the recruitment of inflammatory cytokines and chemokines, scavenging free radicals, and enhancing endogenous enzymatic effects, nonenzymatic antioxidants, chelation of metal ions, and antihyperlipidemic effects [50]. Hosseini et al. reported that thymol inhibited the maturation of dendritic cells and the activation of T-cell proliferation in vitro, and the addition of thymol, γ-terpinene, parafollol, and carvacrol to online feed significantly reduced the expression of the transcription factor nuclear factor κB (NFκB) in the jejunum of broilers [51]. Ashraf et al. reported a significant downregulation of interleukin-6 (IL-6), interferon γ (IFN-γ), and tumor necrosis factor α (TNF-α) expression in broilers following dietary supplementation with thymol [52]. Similarly, Guimaraes et al. demonstrated the efficacy of thymol and carvacrol in three distinct phases of wound healing [53]. In the initial stage, these compounds exhibited a regulatory effect on inflammatory cytokines, oxidative stress, and antimicrobial capacity. Subsequently, they facilitated re-epithelialization, angiogenesis, and the formation of granulation tissue. Finally, they improved collagen deposition and regulated the growth of fibroblasts and keratinocytes.

The above studies indicate that oregano species exhibit potential as anti-inflammatory agents and hold promise for incorporation into formulations aimed at preventing or treating inflammation-related diseases. Nevertheless, further in vivo and clinical investigations are imperative to ascertain the potential toxicological impact of OEO on cellular function prior to its consideration as a viable alternative for inflammation treatment.

### 3.2. Antibacterial Effect of OEO

Antibiotics are frequently employed for the treatment of gastrointestinal and respiratory ailments in livestock animals, such as pigs, poultry, and cattle. However, the dissemination of antimicrobial resistance genes through livestock feces introduces bacteria into the environment. Consequently, there is an urgent need for the development of antibiotic alternatives to mitigate the need for antibiotics [54,55,56,57]. The antimicrobial properties of essential oils derived from herbs and spices, particularly OEO, have been extensively investigated both in vitro and in vivo to assess their potential antibacterial, antiviral, and antifungal activities [58,59] (Table 2). Oregano has been historically utilized at various medicinal dosages for the treatment of respiratory and gastrointestinal disorders, as well as for its antimicrobial properties. In particular, OEO has been recommended for the management of *Candida* infections [60]. The minimum lethal concentrations (MLCs) of *Salmonella* spp. and *L. monocytogenes* in oregano sourced from Italy were determined to be within the range of 0.6–1.2 μL/mL. For oregano originating from Saudi Arabia, the minimum inhibitory concentrations (MICs) for Enteritidis and *L. monocytogenes* were found to be 0.16 and 0.32 mg/mL, respectively. Furthermore, for oregano originating from Iran, the MIC and minimum bactericidal concentration (MBC) for *L. monocytogenes* were determined to be 1.28 and 2.56 mg/mL, respectively, indicating a notable antimicrobial effect of oregano [61,62,63].

In recent years, researchers have found that oregano oil also has the function of regulating rumen microbial community and improving rumen fermentation, which provides a possibility for its application in reducing rumen methane production [64]. A large number of microorganisms live in rumen of ruminants, which decompose cellulose and other sugars in forage through fermentation and produce metabolites such as volatile fatty acids, carbon dioxide, and methane [65]. The production of methane not only wastes energy in feed but is also one of the important greenhouse gases. Therefore, reducing methane production by regulating the rumen microbial community and the fermentation process is an important way to reduce greenhouse gas emissions in animal husbandry. Studies have shown that the active ingredients in oregano oil can inhibit the activity of methanogens and reduce their numbers, thus reducing methane production [14,66].

OEO exhibits a notable capacity to combat biofilm formation through the inhibition of various biofilm-specific mechanisms, including cell–cell interactions, aggregation, motility, the production of extracellular polymeric substances (EPSs), and altered gene expression. This inhibitory effect is observed even at extremely low concentrations, effectively impeding the formation of pathogenic biofilms. The present investigation demonstrated that Greek oregano possesses a robust antimicrobial efficacy comparable to, or potentially surpassing, that of oregano sourced from other countries, as previously reported [67]. In their study, Rupasinghe et al. evaluated the mechanism by which carvacrol inhibits *Streptococcus pyogenes* biofilms [68]. The researchers determined that carvacrol exhibited a minimum biofilm inhibitory concentration (MBIC) of 125 μg/mL against *Streptococcus pyogenes*. Furthermore, through the use of electron microscopy and confocal microscopic analysis, they observed a dose-dependent inhibition of biofilm formation and a decrease in biofilm thickness. Additionally, carvacrol inhibited biofilm-related virulence factors, including cell surface hydrophobicity. The results of quantitative real-time polymerase chain reaction analysis indicated that the expression of the speB, srtB, luxS, covS, dltA, ciaH, and hasA genes, which are known to be involved in biofilm formation, decreased, indicating the therapeutic potential of carvacrol for treating biofilm-associated streptococcal infection.

### 3.3. Growth-Promoting Effects of OEO

Furthermore, OEO possesses antibacterial, antiviral, antioxidant, and anti-inflammatory properties. Additionally, thymol and carvacrol play a role in modulating the immune response and regulating the gut microbial population, thereby influencing growth and feed utilization in meat chickens. The hepatoprotective properties of these compounds were assessed in hepatotoxicity studies, demonstrating their efficacy in protecting the liver of chickens. Moreover, these compounds have the potential to impact the behavior of laying hens, as well as the composition and thickness of eggshells and the sensory quality of eggs [50,69].

The growth rate of livestock is influenced by their food intake and feed conversion rate. Research has shown that thymol and carvacrol can enhance this growth rate by promoting the stability of microbial populations, activating enzymes, improving digestive system function, and enhancing nutrient absorption [70,71]. Additionally, the inclusion of 1% OEO in feed improved the feed conversion ratio, while dietary supplementation with OEO at a dosage of 600 mg/kg significantly reduced feed intake and improved feed conversion in livestock animals. Strkolcova et al. reported significant increases in the ratio of villi height to crypt depth in the small intestinal wall of rabbits (*p* < 0.01), blood phagocytic activity (*p* < 0.0001), and the presence of lactic acid bacteria in the cecal appendix (*p* < 0.01) and fecal lactic acid bacteria (*p* < 0.05) following the discontinuation of thymol [72]. These findings provide evidence that thymol can have a positive impact on the intestinal health and immune response of rabbits, thereby enhancing the fattening ability of farmed rabbits.

In recent years, there has been a growing consumer preoccupation with meat and its quality, with the color of fresh chicken being deemed a significant determinant for purchase [73,74]. Kim et al. conducted a study illustrating that the incorporation of carvacrol, a natural antioxidant, into poultry feed can enhance the overall quality of poultry products [75]. This supplementation effectively reduces lipid oxidation and microbial contamination in chicken patties stored at low temperatures (0–3 °C), thereby extending the shelf life and improving the quality of poultry meat. A previous study has shown that oregano has the potential to normalize lipid and carbohydrate metabolism in livestock and can be used as a supplement to treat hyperlipidemia and type 2 diabetes in overweight patients [76].

### 3.4. Antioxidant Effects of OEO

Cells produce free radicals or reactive oxygen intermediates as part of their normal metabolic process. OEO emerges as a potent combatant against oxidative stress, a state of imbalance between oxidation and the body’s antioxidant defenses. Oxidative stress refers to an imbalance between oxidation and antioxidant effects within the body and is a detrimental consequence of free radicals and a significant contributor to the processes of aging and disease [77,78]. Numerous investigations have established a direct link between oxidative stress and the progression of illnesses such as Alzheimer’s, Parkinson’s, chronic inflammation, arthritis, cancer, diabetes, and atherosclerosis [79,80,81]. OEO’s ability to neutralize free radicals makes it an effective agent against lipid peroxidation in fatty foods. Its antioxidant properties allow it to be incorporated into poultry feed as a natural food-grade antioxidant, thereby delaying spoilage during storage [82]. By harnessing OEO’s antioxidant potential, we can effectively counter the detrimental effects of oxidative stress and promote overall health and well-being. Antioxidants play a significant role in constraining oxidative stress, with mitochondrial SOD and cytoplasmic GPX serving to impede oxidation and the deterioration of mitochondrial membranes. Multiple studies have demonstrated that the administration of oregano results in a noteworthy increase in the levels of GPX, TAC, and SOD. This increase signifies an enhanced antioxidant capacity and potentially contributes to the observed reduction in MDA levels within the group receiving the oregano supplement [83,84,85].

Carvacrol and thymol, which are the primary antioxidants found in OEO, can adsorb and counteract free radicals, thereby contributing to the equilibrium between oxidants and antioxidants and diminishing indicators of muscle impairment [58,86,87]. Through investigations involving the extraction of fatty acid mixtures from mouse brains, it was observed that OEO effectively hindered the auto-oxidation of polyunsaturated fatty acid esters in these brains [88]. The potent free radical scavenging properties of OEO and thymol contribute to its ability to provide protection against chronic diseases and neurodegenerative diseases, as well as its potential as a preservative in food or nutraceuticals [89,90,91,92,93]. In a study conducted by Demir et al., the therapeutic efficacy of carvacrol in mitigating β-amyloid-induced damage in both in vitro and in vivo models of Alzheimer’s disease was assessed [94]. This study highlighted the anti-acetylcholinesterase, antioxidant, and neuroprotective properties of carvacrol. In the present study, an in vivo experiment was conducted to establish a rat model of Alzheimer’s disease (AD) through bilateral intrahippocampal injection of Aβ1–42. The results revealed that carvacrol significantly increased cell viability and exerted protective effects against oxidative stress. This effect was achieved by effectively preventing Aβ1-42-induced cytotoxicity, LDH release, and elevated levels of MDA and H_2_O_2_ in vitro. Furthermore, carvacrol reversed Aβ1-42-induced alterations in passive avoidance tests, thereby ameliorating memory impairment. Additionally, carvacrol was found to mitigate the Aβ1-42-induced increase in MDA and H_2_O_2_ levels in both in vitro supernatant and in vivo hippocampal samples. However, the in vitro treatments did not elicit any significant alterations in the levels of SOD or Tau peptides. Similarly, the in vivo treatments did not result in any notable changes in the levels of MDA, H_2_O_2_, SOD, CAT, Tau peptides, Aβ1-40, or Aβ1-42 in the serum. These findings suggest that carvacrol has the potential to mitigate neurotoxicity, oxidative stress, and memory impairment induced by β-amyloid protein, suggesting that it is a promising therapeutic intervention for Alzheimer’s disease.

### 3.5. Antiviral Effects of OEO

Since the start of global pandemics of multiple viruses, various herbal species with potential antiviral properties, such as flavonoids, terpenoids, polyphenols, coumarins, alkaloids, thiophenes, and others, have been identified [95]. Bernstein et al. reported that carvacrol plays a crucial role as an antiviral component in human rotavirus (RV). Carvacrol has been demonstrated to inhibit viral diseases in both animals and humans. Additionally, oregano and its phenolic components exhibit antiviral activity against acyclovir-resistant herpes simplex virus type 1 and human respiratory syncytial virus [96]. The natural compound carvacrol found in OEO has been extensively advocated for its antiviral properties, demonstrating efficacy against various viral diseases, including the pandemic H1N1 influenza virus. Moreover, it has been shown to directly target the viral capsid, effectively inhibiting human norovirus within a mere 4-h timeframe [97].

### 3.6. Anticancer Effects of OEO

To date, comprehensive explorations of anticancer mechanisms are lacking. Despite the acknowledged importance of OEO in cancer prevention and treatment, the existing research is insufficient to provide a thorough understanding of its anticancer mechanism. Consequently, further confirmatory investigations are imperative to enable accurate analysis. At the same time, there are still many neoplastic diseases to be solved in animal production, and OEO deserves more attention.

There is a substantial body of evidence supporting the antitumor properties of OEO, as demonstrated by its efficacy in both in vivo and in vitro assays. Jaitak et al. successfully confirmed the antiproliferative effects of OEO in various cancer cell models via multiple pathways [98]. Kozachenko et al. reported a reduction in tumor implantation by a factor of 1.8 and a decrease in tumor size by a factor of 1.5 when OEO was administered [99]. Additionally, the authors found significant inhibition of tumor development in mice, indicating that OEO influences the progression and procession of tumors through the activation and regulation of cellular molecules. Katarzyna et al. reported that OEO decreases glucose uptake in cancer cells, impedes extracellular matrix remodeling, hinders the activity of cell adhesion molecules implicated in cancer progression, and impedes the formation of blood vessels necessary for tumor growth [100]. Consequently, the oil exhibited inhibitory effects on tumor development. Additionally, carvacrol, a constituent of OEO, exhibits weak mutagenic and genotoxic properties at nontoxic doses. Notably, carvacrol alone has the potential to selectively target cancer cells and effectively impede their proliferation, thus representing a targeted therapeutic approach. Meena et al. discovered that carvacrol exhibits anti-inflammatory effects through the reduction of oxidative stress, with a primary focus on the ER and mitochondria [101]. Additionally, it effectively modulates the cell cycle and impedes tumor progression. However, existing evidence indicates that carvacrol also plays a crucial role in inhibiting cell migration, invasion, and angiogenesis in tumor cells. This effect may be attributed to the ability of carvacrol to target key biomarkers and major signaling pathways, such as the PI3K/AKT/mTOR, MAPK, STAT3, and Notch pathways, thereby influencing cell survival and cytotoxicity. The elusive nature of the role of carvacrol in osteosarcoma and its underlying molecular mechanisms were investigated by Liang et al. [102]. Their study revealed that carvacrol inhibited colony formation in U2OS and 143B cells in a concentration-dependent manner. Additionally, carvacrol treatment resulted in increased expression of Bax and decreased expression of Bcl-2. Furthermore, carvacrol treatment suppressed the expression of MMP-9 and inhibited the migration and invasion of 143B and U2OS cells. These findings suggest that the effect of carvacrol on osteosarcoma is mediated through the regulation of the Wnt/β-catenin signaling pathway.

## 4. Conclusions

**Figure 1:**
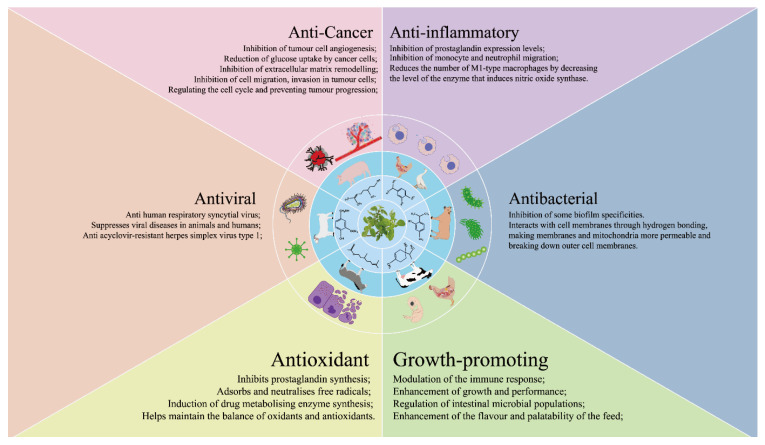
The multifaceted health benefits of oregano essential oil.

This article has provided a thorough examination of the potential utilization of OEO within the domains of animal husbandry and veterinary medicine (Figure 1). OEO shows significant promise as a supplementary component in animal feed, particularly due to its anti-inflammatory, antibacterial, growth-enhancing, antioxidant, and antiviral properties. The aim of this review is to comprehensively analyze the therapeutic application of essential oils extracted from oregano species, with the specific goal of improving animal growth by enhancing intestinal immunity and reducing oxidative stress. In summary, OEO exhibits promising potential as a viable substitute for antibiotics and as a growth promoter in livestock. However, further research is necessary to thoroughly evaluate the exact mechanism through which OEO impacts animal growth performance.

## 5. Future Directions

With food safety and animal welfare issues gradually receiving widespread attention, the animal husbandry and veterinary industry is facing unprecedented challenges and opportunities. In this context, the search for natural, safe, and efficient alternatives to veterinary drugs has become an important research direction for the industry. OEO as a natural extract with antibacterial, antioxidant, and other biological activities, has gradually shown application value in the fields of animal husbandry and veterinary medicine and has broad application prospects. Therefore, we look forward to its future development prospects as follows: First, the mechanism of action of OEO is studied in depth. At present, although OEO has antibacterial, antioxidant and other biological activities, its specific mechanism of action is still unclear. In the future, we need to further explore the specific mechanism of its biological activity to provide more solid theoretical support for the application of OEO in animal husbandry and veterinary medicine. Second, new OEO preparations should be developed. At present, OEO products on the market mainly exist in the form of premixes, and their stability and bioavailability need to be improved. In the future, we can try to develop new preparation methods, such as microencapsulated preparations and nano preparations, to improve the stability and bioavailability of OEO, thereby enhancing its practical application potential. In addition, the application of OEO should be expanded. In addition to its application in traditional livestock disease treatment and growth promotion, we should also actively explore the potential application of OEO for improving the quality of livestock and poultry products and improving animal welfare. For example, to study the impact of OEO on the quality of livestock and poultry meat, its application in improving the anti-stress ability of livestock and poultry should be explored to determine its versatility. Finally, the safety of OEO was evaluated. With the increasing application of OEO in animal husbandry and veterinary medicine, its safety has received increasing attention. To ensure that OEO does not cause potential harm to livestock or humans during use, we need to evaluate its safety, including long-term toxicity, residual problems, and other studies to ensure its safe and effective application in livestock and veterinary practices. In summary, OEO, as a natural extract with various biological activities, has great application potential in the fields of animal husbandry and veterinary medicine. Through in-depth research on its mechanism of action, the development of new preparations, the expansion of application areas, and the strengthening of safety evaluation, we expect to provide new solutions for the sustainable development of the livestock and veterinary industry.

## Figures and Tables

**Table 1:** The top 10 chemical composition of OEO.

No	Compound	%
1	Oxygenated monoterpenes	84.9
2	Carvacrol	70.6
3	Monoterpene hydrocarbons	12.9
4	Linalool	9.7
5	p-Cymene	7
6	γ-Terpinene	2.1
7	Thymol	1.8
8	Sesquiterpene hydrocarbons	1.1
9	Myrcene	1
10	α-Terpinene	1

**Table 2:** Study on antibacterial properties of OEO.

Habitat	Types of Bacteria	Inhibitory Concentration
America	*Candida*	——
Italy	*Salmonella* spp. and *L. monocytogenes*	MLCs = 0.6–1.2 μL/mL
Saudi Arabia	*S. enteritidis* and *L. monocytogenes*	MICs = 0.16 mg/mL and 0.32 mg/mL
Iran	*L. monocytogenes*	MICs = 1.28 mg/mL, MBC = 2.56 mg/mL
China	methanogens	——
Greece	*Streptococcus pyogenes*	MBC = 125 μg/mL

MLCs: minimum lethal concentrations. MICs: minimum inhibitory concentrations. MBC minimum bactericidal concentration.

## Data Availability

The study’s original contributions are included in the article; further inquiries can be directed to the corresponding authors.

## Supplementary Materials

The following supporting information can be downloaded at: https://www.mdpi.com/article/10.3390/ani14111532/s1, Table S1: Main components of the essential oils of oregano species from different regions.
