# Supplementary material for: Oregano Essential Oil in Livestock and Veterinary Medicine
Source: Animals (Basel). 2024 May 22;14(11):1532. doi: 10.3390/ani14111532 (PMC11171306; doi:10.3390/ani14111532)
Supplement: Supplementary file 1 [file animals-14-01532-s001.zip › Supplementary Materials.pdf]

**Table S1.** Main components of the essential oils of oregano species from different regions

| | R1 | R2 | R3 | R4 | R5 |
|---|---|---|---|---|---|
| Longitude | 79.3° | 115.51° | 79.94° | 116.23° | 86.03° |
| latitude | 41.2° | 33.43° | 37.16° | 32.37° | 43.63° |
| Main components | A:85.3%<br>C:5.2% | B:42.9%<br>C: 12.2% | A:75%<br>D: 7.7% | E: 20.8%<br>F:10.2%<br>G:9.8% | H: 32.9%<br>F:17.8%<br>C:10.2% |
| Essential oil yield | 0.7% | 0.3% | 0.3% | 0.3% | 0.1% |

R1: Kunlun Mountains of Hetian; R2: Shangqiu of Henan; R3: Hetian; R4: Anhui; R5: Yili;

A: β-citronellol; B: thymol; C: citronellol acetate; D: trans-geraniol, E: eucalyptol, F: caryophyllene, G: eugenol methylether, H: caryophyllene oxide.
